# A randomized, controlled, single-blinded, multicenter evaluation of the efficacy and safety of a once weekly two dose otic gel containing florfenicol, terbinafine and betamethasone administered for the treatment of canine otitis externa

**DOI:** 10.1186/s12917-018-1627-5

**Published:** 2018-10-11

**Authors:** S B King, K P Doucette, W Seewald, S L Forster

**Affiliations:** 1 Elanco Animal Health, 2500 Innovation Way, Greenfield, IN 46140 USA; 2 Elanco Animal Health Inc., Mattenstrasse 24a, CH-4058 Basel, Switzerland; 3 Elanco Animal Health Ltd., Lilly House, Priestley Road, Basingstoke, Hampshire, RG24 9NL UK

**Keywords:** Otitis externa, Otic gel, Clinical efficacy, Clinical safety, Florfenicol, Betamethasone, Terbinafine

## Abstract

**Background:**

Otitis externa is a common problem in small animal practice. Compliance with daily treatment is a major cause of treatment failure. The hypothesis tested is that a novel otic gel applied to the ear canal twice with a one-week interval is as efficacious as a daily otic suspension in the treatment of canine otitis externa. The study included 286 privately owned dogs with otitis externa.

In this single blinded randomized study, enrolled dogs received either an otic gel containing 1% florfenicol, 1% terbinafine and 0.1% betamethasone acetate twice with a one-week interval or a suspension containing hydrocortisone aceponate, miconazole and gentamicin daily for 5 days. Ears were cleaned with saline prior to administration of the first dose of medication. Dogs were evaluated at day (D) 0, 7, 28 and 56 with an otitis index score (OTIS-3), otic culture and cytology, pain and pruritus, and overall response to treatment (owner and investigator evaluation). Outcome measures were improvement of the OTIS-3 and number of dogs in clinical remission at each time point.

**Results:**

OTIS-3 decreased significantly (*p* < 0.0001) by 63 and 64% for the otic gel and by 63 and 61% for the suspension on D28 and D56 respectively. There was no significant difference between groups at any time point with regard to clinical success, pain, pruritus, overall assessments or otic cytology and culture. The treatment response was considered excellent or good by approximately three quarters of both the clinicians and Owners. Otitis recurrence at D56 was seen in 11% of both groups. Adverse events attributable to the ear medications were not noted.

**Conclusions:**

Administering an otic gel twice at a one-week interval is an effective, safe and convenient way to treat canine otitis externa.

## Background

Otitis externa is one of the more common presenting complaints in small animal practice [1]. The inflammation of the external auditory canal may be due to a number of causes such as hypersensitivities, endocrinopathies, parasites and foreign bodies [2, 3]. More than 50% of patients with atopic dermatitis exhibit otitis externa [4]. Predisposing factors include swimming, other causes of increased moisture in the ear canal or conformational factors such as pendulous pinnae [1, 3]. The consequence of the resultant inflammation and associated decreased ear canal lumen is almost invariably an infection with bacteria and/or yeast organisms [3, 5]. Most topical ear medications on the market therefore contain a combination of antibiotic, antimycotic and anti-inflammatory agents, typically administered once daily [6]. Such products are typically packaged in multi-dose presentations, increasing the potential risk of cross-contamination between ears. In addition to the lack of diagnosis and treatment of the underlying disease, the other major problem treating dogs with otitis is owner and patient compliance [7, 8]. Administration of ear medications into a swollen and often painful auditory canal is a procedure frequently disliked equally by the owner administering the medication and the patient receiving it [8]. In a recent study, formulations requiring infrequent administration and ear cleaning improved the overall Quality of Life for pet owners and their dogs to a greater extent than those requiring daily administration [9].

Increasingly, multi-resistant bacteria make treatment of infections in veterinary medicine more difficult [10–13]. One of the factors recognized to increase the risk for development of such multi-resistant bacterial isolates is repeated exposure to low concentrations of antibiotics [14, 15], such as may occur with poor compliance with administration of a topical antibiotic. Ear medication that reliably maintains an above therapeutic concentration of active ingredients in the ear canal without relying on daily administration by the pet owner could thus be of great benefit to both dogs with otitis externa and their owners.

The study reported here compared the clinical efficacy and safety for otitis externa of an otic gel formulated in a single-use tube administered twice at a one-week interval with a more typical otic suspension administered daily as per the manufacturer’s recommendation, with the objective of demonstrating that the otic gel was non-inferior to the suspension.

## Methods

### Study design

In this randomized, single-blinded, positive-controlled, multicenter field study, dogs received either a viscous otic gel (Osurnia™, Elanco Animal Health, Greenfield, IN, USA) twice with a 1 week interval or an otic suspension (Easotic®, Virbac, Carros, France) daily for 5 days after an initial ear cleaning. Dogs were reevaluated at days 7, 28 and 56 after inclusion. The study was performed in accordance with the VICH GL9 (Good Clinical Practices). This manuscript was prepared after consultation of the checklist of the extension of the CONSORT statement for reporting of non-inferiority trials (www.consort-statement.org/extensions/overview/non-inferiority-and-equivalence-trials; page last accessed October 01, 2018).

### Study objects

Dogs with clinical signs of otitis externa, an otitis index score (OTIS-3) of at least 5 and cytologic evidence of bacteria or yeast were included in the study.

### Inclusion, exclusion and withdrawal criteria

To be eligible for inclusion, dogs were required to be a minimum of 8 weeks of age, of any breed, weight, sex or neuter status. They were excluded due to otic foreign bodies or parasites, if intended for breeding, or if they had been treated with either systemic or topical antimicrobial/antifungals, ciclosporin or anti-histamines within the last 2 weeks, with ear cleaners or analgesic agents within the last week. Cases treated with systemic or topical anti-inflammatories (i.e. corticosteroids or Non-Steroidal Anti-inflammatory Drugs) within the last 28 days were not eligible for inclusion. Staff-owned or animals enrolled in other clinical studies within previous 3 months were not eligible for inclusion.

Similarly, dogs were not permitted into the study if they showed clinical signs of diseases which would interfere with the evaluation of the response. Lastly dogs in which the tympanum was ruptured or still not visible after an initial ear cleaning were also not included in the study. Withdrawal during the study occurred with adverse events that required intervention that could impact the study evaluation, lack of efficacy, administration of prohibited concomitant therapy, owner compliance or any other documented reason.

All study participants were required to sign an Owner Informed Consent prior to enrollment.

### Randomization and blinding

Dogs were randomly allocated to the two treatment groups in a 1:1 ratio in blocks of four using the SAS/STAT® procedure PLAN (SAS, Cary, NC, USA). A separate randomization list was prepared by the statistician for each participating center and provided to the dispensers in the form of sealed numbered envelopes, each containing the treatment allocation according to order of inclusion.

Due to the obvious difference in treatment protocols, Owner blinding was not possible. In each center, a designated person, the “dispenser”, was responsible for the allocation of the dogs to the treatment groups and the administration of the treatments on D0 (and in the group treated twice with otic gel additionally on D7). Prior to each examination by the clinician, the dispenser recorded Owner answers regarding the clinical response, adverse effects, concurrent medications and other clinically relevant information, confirmed the Owner compliance, and prepared the Owner for the visit with the examining clinicians, to assure the continued blinding of the latter and gave relevant discharge instructions.

### Intervention

Prior to inclusion, ear swabs were taken from each affected ear to collect material for culture and cytology. Subsequently, all dogs had their ears cleaned with physiologic saline to remove otic debris and permit visual evaluation of the tympana. Briefly this procedure consisted of filling the ear with warm saline solution, massaging the ear canal and wiping the entrance to the canal out with cotton wool. Dogs could be sedated for ear cleaning, if the ear was too painful to allow cleaning while conscious. Before discharge, each affected ear of dogs in the otic gel group received final formulation of 1 ml of a viscous gel (Osurnia, Elanco Animal Health, Greenfield, IN, USA) containing 1% florfenicol, 1% terbinafine and 0.1% betamethasone acetate. Affected ears of dogs in the control group were treated with one pump (delivering 1 ml) of a suspension (Easotic, Virbac, Carros, France) containing 1.11 mg/ml hydrocortisone aceponate, 15.1 mg/ml miconazole and 1505 I.U./mL of gentamicin. Owners of dogs in the control group were sent home with instructions to administer the otic suspension once daily for four more days. Dogs were reevaluated after 7 days and dogs in the otic gel group were treated again. Ear cleaning was not repeated in either group at D7. These treatment protocols are in accordance with the package inserts for the two products.

### Clinical evaluation

Prior to cleaning of the ears on D0, an otoscopic examination was performed and the OTIS-3 determined. The total score was the sum of the scores for erythema, edema/swelling, erosion/ulceration and exudate, each graded between 0 and 3 (total range 0 to 12) [16]. In bilateral cases, the ear with the higher score was selected to be evaluated throughout for all parameters. If both ears had the same score, the right ear was evaluated throughout. OTIS-3 was determined without further ear cleaning, on D7, D28 and D56.

Overall assessments of response to treatment were performed by the Owners and Investigators at D7, D28 and D56 (excellent, good, moderate or poor; see Table 1). Investigators evaluated pain at each visit using a Numerical Rating Scale ranged 0–3 (0, none; 1, not painful on palpation but spontaneous head shaking; 2, not painful when pinna is raised but painful on palpation of base of ear; 3, painful when pinna is raised). Owners evaluated pain at each visit using a Visual Analog Scale (VAS) with the leftmost end marked ‘The ear is not painful’ and the rightmost end marked ‘The ear is extremely painful’. Pruritus was assessed by Owners at each visit using a VAS with the leftmost corner underlined by the statement “The ear is not itchy” and the rightmost end with the statement ‘The ear is very itchy all the time’.

**Table 1:** Investigator and Owner Overall Assessment Scales

	Investigator	Owner
Excellent	Clinical signs of the ear evaluated during the first examination have completely disappeared	My dog’s ear condition has completely recovered as compared to before treatment
Good	Clear amelioration of the clinical signs of the ear evaluated compared to initial examination	My dog’s ear condition has clearly improved compared to before treatment
Moderate	Slight amelioration of the clinical signs of the ear evaluated compared to initial examination	My dog’s ear condition has responded only slightly to treatment
Poor	Worsening or no change of the clinical signs of the ear evaluated compared to the initial examination	My dog’s ear condition has deteriorated or not changed compared to before treatment

### Microbiological evaluation

At D0, slides were prepared for in-house cytology using each clinic’s standard staining technique. These were examined under low magnification to find an area of interest, with oil then applied and the area was evaluated for presence of microorganisms and inflammatory cells. If the cytology revealed bacteria or yeast, further samples for cytology and culture were sent to a central laboratory (Idexx Laboratories, Ludwigsburg, Germany). Presence of yeast, bacteria and neutrophils was evaluated semi-quantitatively. For neutrophils, a score of 1 was assigned where no neutrophils were seen, 2 for 1–10, 3 for 11–20 and 4 for > 20 within five microscopic fields under × 600 magnification. For yeasts and bacteria, a score of 1 was given when no organisms were seen, few 2 (yeasts < 2, bacteria < 5), moderate 3 (yeasts 3–4, bacteria 5–25) and a high number of organisms recorded as 4 (yeasts > 5, bacteria > 25) based on five microscopic fields under × 600 magnification.

On D28 and D56 additional specimens were obtained for cytology and culture and sent to the central laboratory.

### Minimum inhibitory concentrations

All bacterial and fungal isolates from ear swabs taken both before and after treatment (D28 and D56) from cases treated with the otic gel were supplied to a microbiological testing laboratory (Don Whitley Scientific, Shipley, United Kingdom). The minimum inhibitory concentrations (MIC_50/90_) against all bacterial and fungal isolates for florfenicol and terbinafine was determined using standardized broth microbiology as described by the Clinical and Laboratory Standards Institute (CLSI). Isolates of *Staphylococcus pseudintermedius* and *Staphylococcus aureus* were also screened for methicillin resistance.

### Outcome measures for efficacy

The primary outcome measure was the percentage reduction in OTIS-3 at D28 compared to D0. Secondary outcome measures included the percentage of dogs with an OTIS-3 ≤ 3 (considered a clinical success) at D28 and D56; the percentage reduction in OTIS-3 at D56 compared to baseline; the overall assessments by the Owners and Investigators at D28 and D56; the number of dogs with an OTIS-3 ≥ 5 by D56 (considered relapses if the score at D28 was < 3); the decrease of bacterial or fungal counts on cytology at D28 and D56; the frequency (percentage) of dogs with either a bacteriological or fungal response at D28 or D56 (defined as absence of the microorganism isolated at D0); decrease in pain assessed by the Investigator at D28 and D56; decrease in pain and pruritus assessed by the Owner at D28 and D56; and the speed of response assessed by reduction in OTIS-3 at D7.

### Statistics

An intention to treat (ITT) analysis with the last observation carried forward was performed. Patients completing the study without major protocol deviations were included in a secondary per protocol analysis (PP). Group means were compared between the two groups using an analysis of covariance (ANCOVA) model with the treatment group and baseline response as model effects. The influence of covariates was assessed by a second set of ANCOVA models with body weight, exudate type, chronicity, recurrence, duration, prior treatment, and type of infection as model effects. All calculations were performed using SAS® Version 9.2.2, SAS® Prox Mixed and SAS® Proc Glimmix were used for the ANCOVA and the GLM models (SAS, Cary, NC, USA).

The mean and standard deviation (SD) of the percentage improvement in a previous unpublished pilot study was 66 and 28 respectively. With a non-inferiority margin of 15% (which was considered clinically relevant) and a minimal power of 80%, the appropriate number of study objects was calculated at 100 dogs per group.

### Safety evaluation

Clinical safety of the product was evaluated through the reporting of Adverse Events (defined as any observation in an animal which was unfavorable and unintended and occurred after the use of either product) throughout the study period until D56. Blood samples for hematology and clinical chemistry were collected from all dogs at D0 prior to enrolment and at D28. Samples could be collected at other timepoints at the Investigator’s discretion.

## Results

### Study objects

Thirty first opinion veterinary practices in France, Germany and the United Kingdom enrolled a total of 286 dogs in the study between April and September 2012. All dogs were considered for clinical safety, but one dog was excluded from all demographic and efficacy analyses as the Owner inadvertently retained both copies of the signed consent form. Demographics of the remaining 285 dogs (ITT population) are described herein. One hundred fifty-five males (51 neutered) and 130 female (68 neutered) were included for demographics and efficacy analyses. At the time of enrolment, dogs ranged in age from 10 weeks to 16.5 years with a mean (± SD) of 6.0 (± 3.8) years. The most common breeds represented were Labrador Retrievers (9%), Cocker Spaniels (9%), Golden Retrievers (6%) and Cavalier King Charles Spaniels (6%). Baseline demographics and disease characteristics are summarized by group in Table 2.

**Table 2:** Baseline Demographics

Demographic	Otic Gel	Otic Suspension	*P*-value
Age in years (mean ± SD)	6.2 ± 3.8	5.8 ± 3.7	0.46
Number of Male/Female (%)	55/45	53/47	0.72
Weight in kg (mean ± SD)	21.9 ± 14.4	22.8 ± 14.8	0.62
Bilateral otitis	103 (70%)	101 (74%)	0.51
Ceruminous exudate	108 (73%)	96 (70%)	0.60
Purulent exudate	40 (27%)	41 (30%)	
Acute/subchronic/chronic	42 (28%), 87 (59%), 19 (13%)	30 (22%), 90 (66%), 17 (12%)	0.42
Number of days since onset (mean ± SD)	18.8 ± 22.8	18.6 ± 22.5	0.73
Previously treated	13 (9%)	17 (12%)	0.34
Recurrent	46 (31%)	46 (34%)	0.90
Culture yeast only (%)	25	27	0.89
Culture bacteria only (%)	22	20	
Culture yeasts and bacteria (%)	39	42	

There was no significant difference in any demographic or disease characteristic between groups.

As the results of the ITT and PP analyses were similar, only the results of the ITT analysis are listed and discussed below for efficacy related outcomes.

### Clinical evaluation

The mean OTIS-3 and their standard deviations are listed in Table 3.

**Table 3:** Mean OTIS-3 ± standard deviation of dogs treated with otic gel or otic suspension

Criterion	Visit	Otic gel (*N* = 148)	Otic suspension (*N* = 137)	*P*-value
OTIS-3	D 0	6.8 ± 1.6	6.8 ± 1.6	0.7490
	D 7	3.6 ± 1.7	3.0 ± 1.9	0.0027
	D 28	2.6 ± 2.2	2.6 ± 2.2	0.7992
	D 56	2.5 ± 2.4	2.7 ± 2.4	0.3532

There was a significant improvement in OTIS-3 in both groups after 28 days (*p* < 0.0001). The mean OTIS-3 stayed low until D56 in both groups. The OTIS-3 decreased on average by 62.5 and 63.6% for the otic gel and by 63.4 and 60.5% for the otic suspension on D28 and D56 respectively. Using the non-inferiority margin of 15%, the 95% confidence interval for the difference in percent reduction at D28 (otic suspension minus otic gel) had to be completely below 0.15 × 63.4% i.e. 9.5%. The Confidence Interval was calculated to be − 6.1 to 7.9%, so the primary endpoint of non-inferiority at D28 was concluded.

At D7, there was a significant difference between the two groups for total OTIS-3 (*p* = 0.0027) and the percentage reduction in OTIS-3 (*p* = 0.0003) in favour of the otic suspension. However, no significant difference was seen in the proportion of cases considered a clinical success by the Investigator at D7 (Table 4).

**Table 4:** Number (Percentage) of dogs reported as clinical treatment success^a^ when treated with an otic gel or an otic suspension

Visit	Otic gel	Otic suspension	*P*-value
D7	80 (54%)	84 (61%)	0.2319
D 28	109 (74%)	98 (72%)	0.6926
D 56	106 (72%)	91 (66%)	0.3705

^a^Clinical success was defined as an OTIS-3 ≤ 3

Approximately three quarters of clinicians and Owners reported a good to excellent response of otitis externa to the otic gel administration after D28 (Table 5), which was not significantly different to the suspension.

**Table 5:** Clinician (owner in parentheses) overall assessment of treatment response (in %)

	D 28		D 56	
Response assessment	Gel	Suspension	Gel	Suspension
Excellent	29 (34)	31 (42)	39 (44)	38 (49)
Good	45 (49)	40 (42)	32 (36)	28 (24)
Moderate	19 (10)	20 (11)	18 (7)	22 (17)
Poor	7 (5)	8 (4)	11 (12)	12 (9)
Missing	1 (1)	1 (1)	1 (1)	1 (1)
p-value	0.9901 (0.2692)		0.6169 (0.7980)	

At D56, there were no differences between the group treated with the gel and that with the suspension in owners and investigators overall assessment scores. No significant differences in pain or pruritus scores were observed between the two groups at D28 or D56 (*p* > 0.1860; Table 6).

**Table 6:** Mean scores of all dogs for cytology scores, microbiological responses, pain and pruritus scores

	D0			D7			D28			D56		
	Gel	Suspension	*p*-value	Gel	Suspension	*p*-value	Gel	Suspension	*p*-value	Gel	Suspension	*p*-value
Cytology (Mean +/− SD)												
Bacteria	1.7 +/− 0.8	1.7 +/− 0.8	0.7479	n/a	n/a	n/a	1.1 +/− 0.9	0.9 +/− 0.7	0.2160	1.1 +/− 1.0	0.9 +/− 1.0	0.2233
Yeasts	2.1 +/− 0.8	2.2 +/− 0.8	0.3957	n/a	n/a	n/a	1.3 +/− 0.8	1.5 +/− 0.8	0.1077	1.5 +/− 0.9	1.5 +/− 0.9	0.4040
Neutrophils	1.2 +/− 0.4	1.3 +/− 0.7	0.6431	n/a	n/a	n/a	0.5 +/− 0.7	0.4 +/− 0.8	0.3488	0.5 +/− 0.7	0.4 +/− 0.9	0.2335
Bacteriological Response (%)	n/a	n/a	n/a				60	60	1.0000	49	55	0.4501
Fungal Response (%)	n/a	n/a	n/a				76	69	0.3318	65	55	0.1824
Investigator Pain Score (est. +/− SE)	1.77 +/− 0.07	1.69 +/− 0.07	0.4406	0.45 +/− 0.05	0.55 +/− 0.05	0.2371	0.38 /− 0.06	0.46 +/− 0.06	0.2967	0.42 +/− 0.06	0.54 +/− 0.07	0.1860
Owner Pain VAS (est. +/− SE)	31.4 +/− 2.8	31.1 +/− 2.9	0.9499	8.3 +/− 0.8	6.8 +/− 0.7	0.1437	3.9 +/− 0.5	3.3 +/− 0.4	0.3984	3.8 +/− 0.5	4.1 +/− 0.6	0.7676
Owner Pruritus VAS (est. +/− SE)	50.2 +/− 3.2	52.5 +/− 3.4	0.6334	10.8 +/− 1.1	7.8 +/− 0.8	*0.0232*	5.0 +/− 0.6	4.3 +/− 0.6	0.423	5.1 +/− 0.8	5.2 +/− 0.8	0.9208

*Est.* Estimated, *S.E.* Standard error, *VAS* Visual analog scale, *SD* Standard deviation; Entries in *italic* font are statistically significant

However, the owners reported a significant difference in pruritus at D7, in favor of the suspension (*p* = 0.0232). Relapses were recorded at D56 in 11 dogs (11%) treated with the otic gel, and in 10 dogs (11%) treated with otic suspension; there was no significant difference between the groups. Subsequent exploratory analyses did not identify any difference in clinical success at any timepoint between groups dependent on the weight of the dog (p > 0.18 across all weight bands and time points), chronicity of disease (*p* > 0.1727), recurrence (*p* > 0.0947), previous treatment (*p* > 0.2025) or type of exudate (*p* > 0.0638).

### Microbiological evaluation

On D0, 40% of the overall study population had positive otic cultures for both bacteria and yeast, 26% had only yeast, 21% only bacteria and the remainder were culture negative; staphylococci of the *Staphylococcus pseudintermedius* group were most frequently cultured (47%), followed by *Pseudomonas aeruginosa* (11%), streptococci (11%) and enterococci (5%); in 18% of the dogs more than one bacterial species was identified. Of the proportion of the study population in which yeast was cultured, the most frequently identified organism was *Malassezia pachydermatis* (96%). There was no difference in distribution between groups for either bacteria or yeast species at D0.

The cytology counts for bacteria, fungi and neutrophils decreased in both groups, with no significant difference seen between groups (Table 6).

The overall bacteriological response (i.e. elimination of pathogens identified at baseline culture) as assessed by repeat of bacterial culture, was 60% and 49% for the gel at D28 and D56 respectively, compared to 60% and 55% for the otic suspension. For the fungal response, 76% and 65% of cases treated with the otic gel had responded at D28 and D56 respectively, in comparison to 69% and 55% for the otic suspension. None of these were significantly different (Table 6).

When clinical success (OTIS-3 ≤ 3 at D28) was considered for cases with different bacterial species, no significant differences were found between the products for any species. For *S. pseudintermedius*, the clinical success rate was 75% and 72% at D28 for the otic gel and the suspension respectively. For *P. aeruginosa*, the clinical success rates were 47% and 50% respectively for the otic gel and the otic suspension at D28.

### Minimum inhibitory concentrations

The MIC data demonstrated that florfenicol was active against all bacterial groups, with MIC_50_ in the range 2 to 16 μg/ml except for *P. aeruginosa* (> 128 μg/ml). Due to low numbers of isolates for other pathogens, the MIC_90_ could only be calculated for *S. pseudintermedius* (8 μg/ml), *Streptococcus canis* (2 μg/ml) and *P. aeruginosa* (> 128 μg/ml)_._ One hundred and two (102) *S. pseudintermedius* isolates were identified, of which 2 were identified as methicillin resistant on the basis of oxacillin zone diameter. Of 4 *Staphylococcus aureus* isolates, 2 were identified as methicillin resistant on the basis of cefoxitin zone diameter. All isolates identified as methicillin resistant exhibited florfenicol MICs within the same range as methicillin susceptible isolates of the corresponding species.

The only fungal species with greater than 10 isolates was *M. pachydermatis*. The MIC for terbinafine was in the range 0.125 to > 64 μg/ml, with an MIC_90_ of 2 μg/ml.

In 59% of cases, there was no change in the MIC for florfenicol between baseline and D28 of any organism identified in an individual ear. In 28% of cases, there was an increase of MIC by one dilution, with a decrease by one dilution in the remainder (13%). For terbinafine, there was no change in MIC between baseline and D28 for 55.5% of Malassezia cases, with 28% having an increase by one dilution, and 16.5% having a decrease by one dilution.

### Safety evaluation

A total of 100 clinical signs relating to 80 adverse events were recorded in 30 of the dogs treated with the otic gel and 32 of the dogs treated with the suspension. These are summarized by System Organ Class in Table 7.

**Table 7:** Clinical signs seen with administration of otic gel or suspension in dogs with otitis externa

System Organ Class	Otic Gel (*n* = 148)	Otic Suspension(*n* = 138)	*P*-value
Behavioral disorders	0	2	0.2328
Blood and lymphatic system disorders	3	0	0.2509
Digestive tract disorders	15	10	0.4315
Ear and labyrinth disorders	7	4	0.5516
Eye disorders	1	6	0.0617
Hepato-biliary disorders	1	0	1.0000
Mammary gland disorders	0	1	0.4825
Musculoskeletal disorders	1	3	0.3585
Neurological disorders	1	0	1.0000
Renal and Urinary disorders	0	1	0.4825
Reproductive system disorders	0	1	0.4825
Respiratory tract disorders	0	1	1.0000
Skin and appendages disorders	16	18	0.6105
Systemic disorders	5	2	0.4551
Any clinical sign	51	49	0.9204

Cutaneous signs, such as generalized pruritus (rather than local to the ear) were the most common and observed in approximately one third of dogs showing adverse events, followed by slightly less dogs showing gastrointestinal signs. The majority of these adverse events were likely related to an underlying disease process such as atopic dermatitis. There was no difference in occurrence for any adverse event between groups (*p* = 0.9204). There was no recognizable association between treatment and adverse events.

Three dogs treated with the otic gel showed clinically severe adverse events, none of those was considered to be related to medication due to the lack of temporal association and clinical signs observed. One dog had intestinal obstruction by peach stones 7 weeks after treatment, one a septic abdomen 6 weeks after treatment and the last one underwent exploratory surgery 7 days after inclusion in the study and showed gastrointestinal neoplasia. The first dog recovered uneventfully after surgery, the other two dogs were euthanized.

No changes in body weight, or clinically significant changes from baseline were seen in hematology or serum chemistry variables for either the gel or the suspension. There were no clinical pathology changes which could be considered of either hyper- or hypoadrenocorticism.

## Discussion

In this study, in a population of dogs displaying typical characteristics of otitis externa, administration of an otic gel twice 1 week apart demonstrated similar efficacy and clinical safety to treatment with a conventional daily suspension for most endpoints. Only for the secondary endpoint speed of response (in terms of absolute and percentage reduction in OTIS-3 and Owner pruritus VAS) was the daily treatment significantly better than the otic gel. However, in another recently published study [9] using the otic gel, the percentage improvement in pruritus was considered to be better with the gel in comparison to a different daily treatment to that used in the current study. In that study, the comparator product contained a more potent corticosteroid (mometasone furoate) but had a smaller daily dose volume than the comparator in the current study. The smaller dose volume may not allow for sufficient contact between the product and the entire ear canal to rapidly reduce pruritus. Unlike the current study, Noli et al. [9] found that no difference in percentage improvement of OTIS-3 at D7 was seen between the gel and the comparator daily treatment. However, in that study, the gel provided significantly greater percentage improvements in cytology scores at Day 7 and Day 28. This suggests that the gel is capable of producing significantly faster responses to treatment than some daily treatments.

Approximately one third of dogs had recurrent otitis externa and in a little more than 10% the otitis was defined as chronic with those numbers comparatively lower than reported in one large epidemiological study [2]. This discrepancy may be explained by the fact that studies are frequently published by referral institutions and in contrast the practices involved in this study were first opinion. As in other studies, most dogs were presented with bilateral otitis, although the number of dogs with unilateral otitis externa was higher in this study than in reported studies (30% versus 6–7%) [2, 17], again probably reflecting the early presentation more typical for first opinion practice.

Staphylococci were the most common bacteria cultured from the dogs in this study, which is in accordance with other publications [17–20]. *P. aeruginosa* was cultured in a smaller number of dogs, also similar to other publications [17, 20]. In contrast with other reports [21, 22], the smaller number of dogs infected with *Pseudomonas* in this study probably reflects early presentation and first opinion practice. A large number of dogs showed *Malassezia* organisms, either with or without concurrent bacteria. These findings are in concordance with other studies [2, 3, 17], although in most publications, the exact number of dogs showing *Malassezia* organisms only versus concurrent bacterial and yeast infection was not stated.

The OTIS-3 scale used in this study was validated in a recent study [16]. A total score of ≥4 differentiated healthy from diseased ears with a specificity of 100% and a sensitivity of over 90%. Based on that publication, an OTIS-3 ≥ 5 was a criterion of inclusion in this study and an OTIS-3 of ≤3 was considered an ear in clinical remission. Utilizing this score, slightly less than three quarters of the dogs included in this study were in clinical remission after two treatments with the gel and approximately two thirds after five treatments with the suspension. This compares favorably to a number of other studies. In a study using a zinc-acetic acid ear cleaner for 14 days, 25% of the dogs with otitis externa due to *M. pachydermatis* had a clinical score indicating clinical remission [23]. In another study, 140 dogs were treated with either a marbofloxacin/dexamethasone/clotrimazole suspension daily or with miconazole/polymixin B/prednisolone daily for 7 to 14 days. The cure rate by day 14 was 58 and 41% respectively, defined by absence of erythema or ulceration, pruritus, pain and smell [24]. The latter study was also conducted in first opinion practices. A different method of scoring was used in each of these 3 studies making direct comparison difficult. One small study treating 12 dogs with twice daily ear cleaner and ticarcillin for up to 1 month had a better success rate, all but one dog responded well [25]. However, this study was not controlled, the dogs were referred for chronic otitis and treated much more aggressively including repeated procedures of ear flushing under anaesthesia. Owners used an ear cleaner twice daily and the ear medications were used for more than 2 weeks.

Cytology of the ear swabs showed a significant reduction of yeast and bacteria during treatment in both groups. On D28, approximately two thirds of the population and on D56, approximately half of the population showed no or few bacteria cytologically. Only a few studies have evaluated cytology subsequent to the treatment of otitis externa, and the methods differed in each study, thus comparisons are difficult. In a study using a zinc-acetic acid ear cleaner for 14 days, 58% of the dogs with otitis externa due to *M. pachydermatis* had few or no yeast after 14 days [23]. In a study of dogs with *Malassezia* otitis treated with either miconazole/ dexamethasone/saline or the same solution with an added chelating agent the clinical scores and cytological scores for yeast organisms decreased significantly, but it was not stated how many dogs were negative on cytology and in remission clinically [26]. The apparent increase in bacterial cytological counts between D28 and D56 is likely to be as a result of recolonization of the ear canal by the normal flora.

Determination of MICs in this study demonstrated that most routinely identified pathogens associated with otitis externa are highly susceptible to both florfenicol and terbinafine. It was noted that despite the higher MIC, the clinical success rate in cases with *P. aeruginosa*, whilst lower than clinical success rates for *S. pseudintermedius,* was similar in both groups at D28. This is likely because topically applied antibiotics will more often than not exceed MICs established for systemic administration, even for apparently resistant organisms [27]. Although changes in MIC were observed in approximately 40% of cases at D28 compared to baseline, these were never by more than one dilution. It is considered that this is due to normal variation in the reproducibility of MIC data, as the changes were in both directions from baseline. Thus, despite the persistence of the antimicrobials within the ear canal [28], no evidence of a change in susceptibility of the target organisms was observed in this study.

Adverse events were seen in approximately 15% of the dogs in this study. However, it is important to note that adverse events were classified as any unfavorable and unintended event during or after administration of the medications, whether or not considered to be product related [29]. Adverse events by and large could be divided into two major groups. Firstly, clinical signs such as pruritus, dermatitis, conjunctivitis, otitis, or pyoderma which were most likely related to the primary cause of the otitis externa; and secondly clinical signs such as vomiting or diarrhoea, which were unlikely to be related to the underlying cause of otitis. Half of all dogs with allergic skin disease are presented with otitis externa [4, 30]. Allergic skin disease frequently waxes and wanes depending on the offending allergens and is associated with pruritus and secondary infections [4, 30], thus it is not unreasonable to assume that increased generalized pruritus in a patient is more likely due to its underlying allergy than due to the application of an otic topical. It was not the goal of the study to determine the underlying predisposing causes and as such no detailed history of other clinical signs was recorded, which would have been useful to determine whether indeed these adverse events were previously present. The second large group of adverse events included signs such as abdominal pain, pseudopregnancies, arthritis and vomiting. These clinical signs were most likely not related to the otitis externa, its primary cause or its treatment. Arthritis is a common complaint of older dogs [31] and the age of dogs included in this study was up to 16 years with a mean of 6.0 years. Gastrointestinal signs such as vomiting and diarrhea are frequently seen in dogs [32, 33], their cause is often unknown and they often resolve without specific therapy. Only three dogs (all treated with the otic gel) were withdrawn from the study due to serious adverse events, which were gastrointestinal neoplasia, obstruction due to intestinal foreign bodies and a septic abdomen 6 weeks after enrolment. These are highly unlikely to be related to the treatment of the otitis externa. Overall, adverse effects were considered not to be related to treatment, but rather to underlying or concurrent diseases and no clinically relevant changes were seen in any serum biochemistry or hematology parameter.

## Conclusion

The non-inferiority of the otic gel to the otic suspension as a reference product was demonstrated following two administrations of the gel at a one-week interval. Clinical signs of the dogs with otitis externa improved similarly in the two groups, independent of the nature and chronicity of the infection. In both groups there was a significant decrease in otic scores and microorganisms as well as a rapid improvement in pain and pruritus scores. The otic gel was well tolerated in a wide range of ages and breeds of dog and thus offers a safe and effective treatment alternative for dogs with otitis externa with the added benefit of requiring only two administrations 1 week apart, providing the potential for improved compliance with the treatment regimen.

## Abbreviations

ANCOVA: Analysis of covariance
CLSI: Clinical and Laboratory Standards Institute
D: Day
ITT: Intent to treat
MIC: Minimum inhibitory concentration
OTIS-3: Otitis index score
PP: Per protocol
SD: Standard deviation
